# Moderate Exercise Improves Experimental Cancer Cachexia by Modulating the Redox Homeostasis

**DOI:** 10.3390/cancers11030285

**Published:** 2019-02-28

**Authors:** Riccardo Ballarò, Fabio Penna, Fabrizio Pin, Mari Carmen Gómez-Cabrera, José Viña, Paola Costelli

**Affiliations:** 1 Department of Clinical and Biological Sciences, Experimental Medicine and Clinical Pathology Unit, University of Torino, 10125 Torino, Italy; riccardo.ballaro@unito.it (R.B.); fabio.penna@unito.it (F.P.); fpin@iu.edu (F.P.); 2 Interuniversity Institute of Myology, 61029 Urbino, Italy; 3 Department of Anatomy and Cell Biology, Indiana University School of Medicine, Indianapolis, IN 46202, USA; 4 Department of Physiology, Freshage Research Group, University of Valencia, CIBERFES, INCLIVA, 46010 Valencia, Spain; gomez@uv.es (M.C.G.-C.); jose.vina@uv.es (J.V.)

**Keywords:** muscle wasting, oxidative stress, autophagy, chemotherapy, mitochondria

## Abstract

Cachexia is a debilitating syndrome that complicates the management of cancer patients. Muscle wasting, one of the main features of cachexia, is associated with hyper-activation of protein degradative pathways and altered mitochondrial function that could both result from impaired redox homeostasis. This study aimed to investigate the contribution of oxidative stress to cancer-induced cachexia in the presence or in the absence of moderate exercise training. Mice bearing the colon C26 carcinoma, either sedentary or exercised, were used. The former showed muscle wasting and redox imbalance, with the activation of an antioxidant response and with upregulation of markers of proteasome-dependent protein degradation and autophagy. Moderate exercise was able to relieve muscle wasting and prevented the loss of muscle strength; such a pattern was associated with reduced levels of Reactive Oxygen Species (ROS), carbonylated proteins and markers of autophagy and with improved antioxidant capacity. The muscle of sedentary tumor hosts also showed increased levels of molecular markers of mitophagy and reduced mitochondrial mass. Conversely, exercise in the C26 hosts led to increased mitochondrial mass. In conclusion, moderate exercise could be an effective non-pharmacological approach to prevent muscle wasting in cancer patients, decreasing muscle protein catabolism and oxidative stress and preserving mitochondria.

## 1. Introduction

Cancer cachexia is a complex syndrome characterized by body weight loss, muscle wasting and metabolic alterations that occurs in 50–80% of advanced malignant tumors, accounting for about 20% of cancer patient deaths [1]. Muscle wasting is one of the main clinical events and negatively correlates with anticancer treatment tolerance and effectiveness, resulting in reduced patient survival [2,3]. Muscle protein depletion is driven by tumor and host-derived humoral mediators that activate muscle proteolysis, with particular emphasis on proteasome-dependent degradation and autophagy [4]. Beyond protein breakdown, hypoanabolism and mitochondrial alterations may also contribute to cancer cachexia [5,6,7]. Notably in the skeletal muscle, a reduced number of mitochondria and impaired mitophagy correlate with loss of mass and function in both experimental and human cancer cachexia [5,8]. 

Increased muscle protein breakdown rates as well as impaired mitochondrial mass and function could be due, partially at least, to oxidative stress [9]. This latter reflects the alteration of the redox homeostasis in favor of pro-oxidant species, leading to protein, lipid and DNA damage and impaired intracellular signaling [10]. In this regard, a correlation between muscle wasting and increased Reactive Oxygen Species (ROS), lipid peroxidation products and protein carbonylation has been found in cachectic animals [11,12,13], and is associated with increased muscle protein degradation [13]. Notably, the direct effect of oxidative stress on proteolysis has been demonstrated in murine myotubes exposed to FeSO_4_ and H_2_O_2_, resulting in the overexpression of components of the ubiquitin-proteasome pathway and in increased proteasome chymotrypsin-like enzymatic activity [14]. Increased oxidative stress coupled with inflammation and protein hypercatabolism has also been reported in cachectic cancer patients [15]. Moreover, a growing body of evidence suggests that ROS contribute to regulate autophagy by modulating several signaling pathways such as those dependent on PI3K/Akt/mTORC1 and p38/MAPK/p53 and by impinging on both AMPK and FoxO activity [16,17]. As a negative feedback, autophagy activation may reduce oxidative stress and, on the other hand, its dysregulation could lead to increased ROS production [17]. The cellular redox balance is significantly affected by mitochondria, which are both the source and the target of ROS [18]. In cachectic tumor-bearing mice the pro-oxidant species directly damage mitochondria, leading to enhanced ROS production [18] and to increased mitophagy, likely impacting on muscle mitochondrial abundance [19]. 

Several antioxidant enzymes, including CuZn SOD, catalase and glutathione peroxidases (GPxs) prevent intracellular ROS accumulation, limiting cell damage [10]. In cancer cachexia, the antioxidant enzymes have been reported to be either upregulated, although not enough to maintain the redox balance [11,15], or downregulated [12,20], further favoring the oxidative stress.

At present, there are no effective treatment for cancer cachexia. Many studies have suggested that exercise could represent a promising non-pharmacological approach to relieve cancer-induced alterations in the host [21]. Notably, cancer patients performing exercise show reduced fatigue, improved muscle strength and improved quality of life [22,23,24]. Different exercise protocols have been shown to attenuate body and muscle wasting in experimental cancer cachexia [25,26], reducing proteasome-dependent protein degradation and restoring physiological levels of autophagy [25]. Moreover, exercise modulates the redox balance, depending on training timing and intensity. While exhaustive-sporadic exercise induces both muscle damage and oxidative stress [10], moderate and continuous exercise results in adaptive metabolic changes leading to increased antioxidant capacity [27]. Additionally, exercise could indirectly decrease the intracellular ROS production by attenuating systemic inflammation [28]. 

While the involvement of oxidative stress in C26-induced cachexia was previously demonstrated [29], the effectiveness of exercise in modulating muscle redox capacity was poorly investigated. The present study fills this gap by evaluating how moderate exercise can impinge on the skeletal muscle of C26-bearing mice, analyzing the muscle redox potential, the levels of ROS-derived products and the contribution of the antioxidant systems and autophagy to the redox balance. Moreover, the occurrence of muscle oxidative stress and the effects of exercise were also evaluated in chemotherapy-treated tumor-bearing mice. Indeed, previous studies suggested that cachexia was worsened by the administration of anticancer drugs, and that such a phenotype could be improved by exercise [30]. The results show that moderate exercise is able to relieve muscle wasting and oxidative stress in tumor hosts, while only the former is improved in the presence of chemotherapy.

## 2. Results

**Figure 1:**
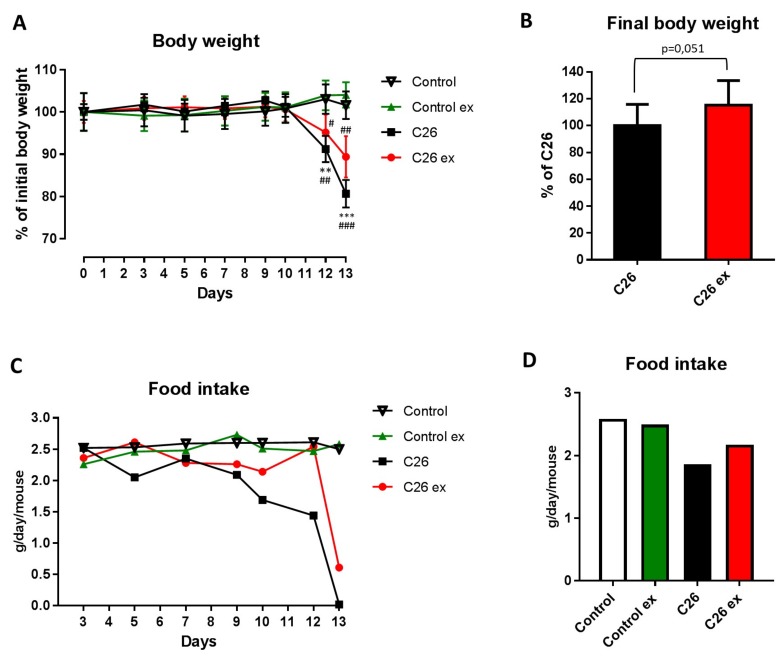
Exercise relieves body wasting and anorexia in tumor-bearing mice. Body weight change (**A**) of control (*n* = 5), control exercised (control ex; *n* = 6) and tumor-bearing mice either sedentary (C26; *n* = 8) or exercised (C26 ex; *n* = 8). Final body weight (**B**) of tumor-bearing mice either sedentary (C26; *n* = 8) or exercised (C26 ex; *n* = 8). Food intake change (**C**) and cumulative food intake (**D**) of control (*n* = 5), control exercised (control ex; *n* = 6) and tumor-bearing mice either sedentary (C26; *n* = 8) or exercised (C26 ex; *n* = 8). Body weight change (panel c) is expressed as percentage of initial body weight (means ± SEM) whereas final body weight (body weight–tumor mass; panel d) is expressed as percentage of C26 (means ± SD). Food intake is expressed as grams/day/mouse (panel c) or average grams/day/mouse (panel d). For panel c and d, the lack of error bars is due to mice housing grouped in cages, not allowing the measurement of individual mouse food intake. Significance of the differences: ** *p* < 0.01, *** *p* < 0.001 vs. control; ^#^ *p* < 0.05, ^##^ *p* < 0.01, ^###^ *p* < 0.001 vs. control ex.

**Figure 2:**
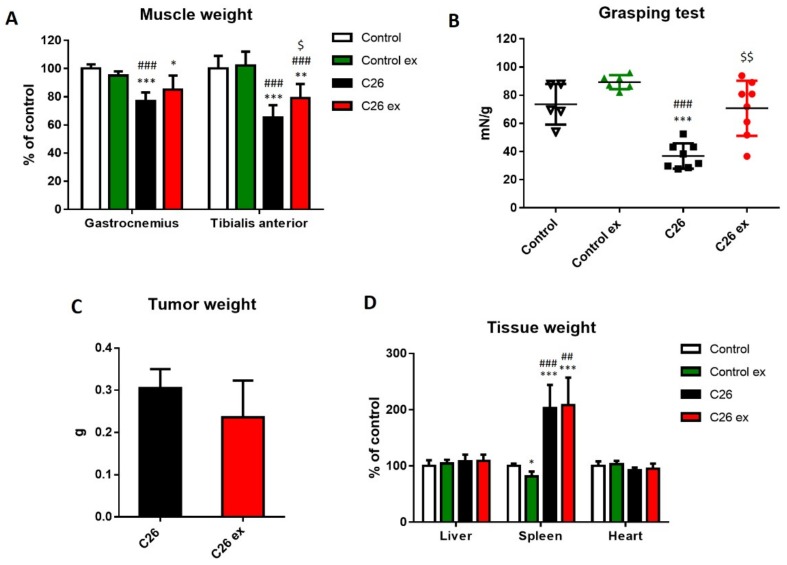
Exercise partially prevents the loss of muscle mass and function in tumor-bearing mice. Muscle weight (**A**), grip strength test (**B**) and tissue weight (**C**) of control (*n* = 5), control exercised (control ex; *n* = 6) and tumor-bearing mice either sedentary (C26; *n* = 8) or exercised (C26 ex; *n* = 8). Tumor weight (**D**) of sedentary (C26; *n* = 8) or exercised mice (C26 ex; *n* = 8). Muscle and tissue weight (means ± SD) are expressed as percentage of control. Grip strength data (means ± SD) are expressed as the ratio of unit force (mN) and initial body weight (g). Tumor weight (means ± SD) is expressed in grams (g). Significance of the differences: * *p* < 0.05, ** *p* < 0.01, *** *p* < 0.001 vs. control; ^##^ *p* < 0.01, ^###^ *p* < 0.001 vs. control ex; ^$^ *p* < 0.05, ^$$^ *p* < 0.01 vs. C26.

Mice bearing the C26 tumor showed a marked loss of body weight as compared to controls (Figure 1A). Body weight loss in the C26 hosts (Figure 1A) was slightly, but with borderline significance (*p* = 0.051), attenuated by exercise (Figure 1A,B), while no differences could be observed between sedentary and exercised controls (Figure 1A). As for food intake, the data presented in Figure 1C,D suggested that mice bearing the C26 tumor reduced their food intake and that exercise could partially protect from this alteration, also inducing a 2-day delay in food intake reduction (Figure 1C). However, since mice were housed grouped in cages, standard deviation and statistical significance among groups could not be calculated. Gastrocnemius and tibialis anterior weight, as well as muscle strength, were lower in the C26 hosts than in control mice (Figure 2A,B). Exercised C26-bearing animals were partially protected from the loss of muscle mass and strength (Figure 2A,B). Such beneficial effect was achieved without significant changes in tumor mass (Figure 2C). In both exercised and sedentary tumor-bearing mice, spleen weight increased whereas liver and heart mass were not affected (Figure 2D). Exercise did not induce any significant change in healthy animals (Figure 1 and Figure 2), the only exception being spleen mass that was reduced as compared to sedentary controls (Figure 2D).

**Figure 3:**
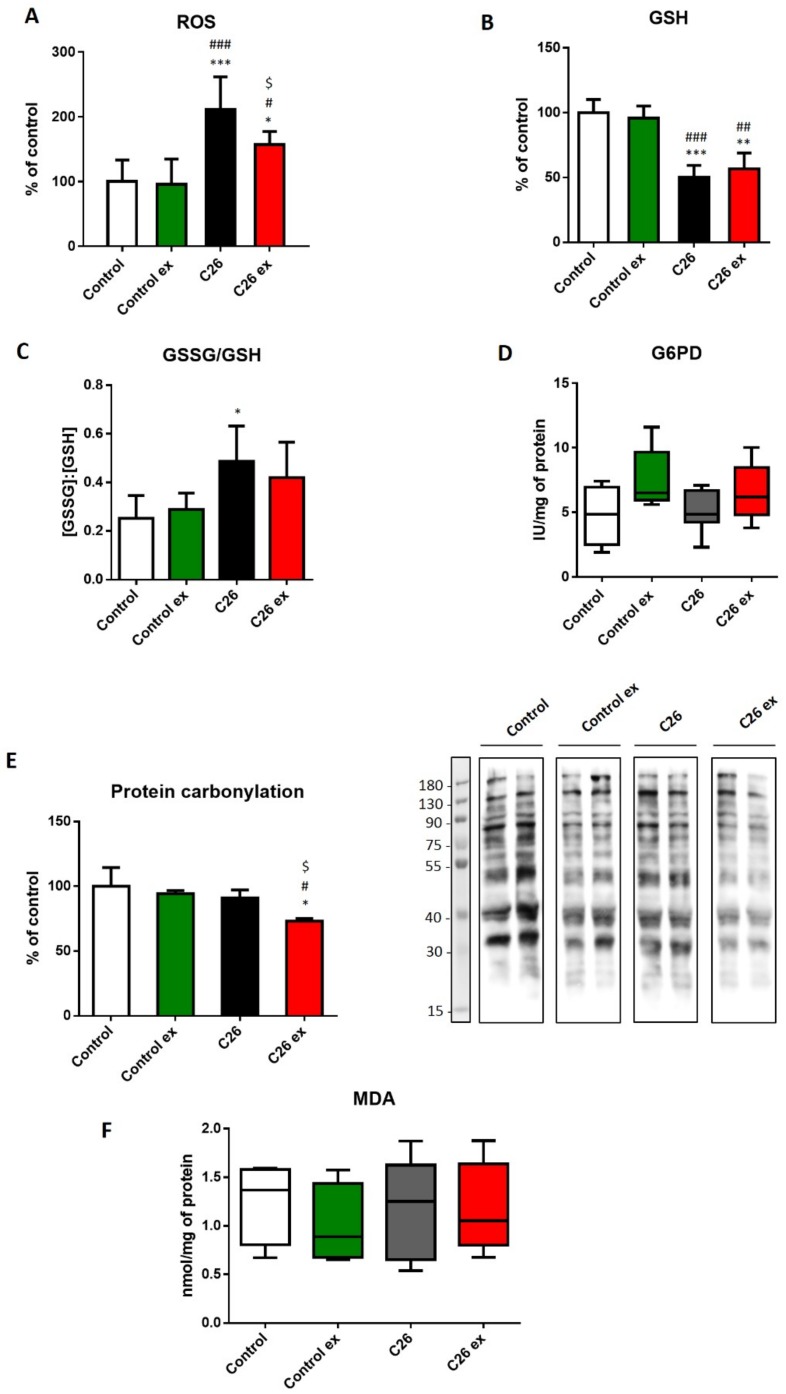
Tumor-bearing mice show a redox imbalance that is partially corrected by exercise. ROS levels (**A**), GSH levels (**B**), GSSG/GSH ratio (**C**), G6PD enzymatic activity (**D**), protein carbonylation (**E**; representative blot and quantification) and MDA levels (**F**) of control (*n* = 5), control exercised (control ex; *n* = 6) and tumor-bearing mice either sedentary (C26; *n* = 8) or exercised (C26 ex; *n* = 8). ROS, GSH and protein carbonylation levels (means ± SD) are expressed as percentage of control. GSSG/GSH data (means ± SD) are expressed as the ratio of GSSH and GSH concentrations. G6PD activity (median and min to max whiskers) is expressed as specific activity. MDA levels (median and min to max whiskers) are expressed as nmol/mg protein. Significance of the differences: * *p* < 0.05, ** *p* < 0.01, *** *p* < 0.001 vs. control; ^#^ *p* < 0.05, ^##^ *p* < 0.01, ^###^ *p* < 0.001 vs. control ex; ^$^ *p* < 0.05 vs. C26.

Aiming to investigate the oxidative balance in the skeletal muscle of trained and sedentary tumor-bearing mice, we assessed ROS levels and both GSH content and GSSG/GSH ratio (Figure 3A,B) as a measure of muscle oxidative stress. C26 hosts showed increased ROS levels as compared to control animals, together with reduced GSH content, resulting in increased GSSG/GSH ratio (Figure 3A–C). For the sake of correctness, it must be quoted that the GSSG/GSH ratio in the present study is higher than that usually reported in the literature [13,31]. Such discrepancy could result from tissue manipulation and assay procedure that could have contributed to enhanced GSH oxidation; however, being all samples processed at the same time, the comparison among groups should not be affected. Since the enzymatic reduction of GSSG to GSH requires NADPH, we investigated if the decrease in GSH levels was associated with impaired G6PD activity, this enzyme being the main NADPH source. G6PD enzymatic activity did not change among the four experimental groups, although a trend towards increase could be observed in exercised animals (Figure 3D). Despite the evidence that oxidative stress was indeed occurring in the muscle of sedentary tumor hosts, the levels of oxidized products such as carbonylated proteins and MDA remained comparable to controls (Figure 3E,F). Exercise in the C26-bearing mice was associated with decreased ROS levels (Figure 3A) and protein carbonylation (Figure 3E), without changes in GSH content, GSSG/GSH ratio and MDA levels (Figure 3B,C,F). On the other hand, exercise did not modulate any marker of oxidative stress in control mice (Figure 3). 

**Figure 4:**
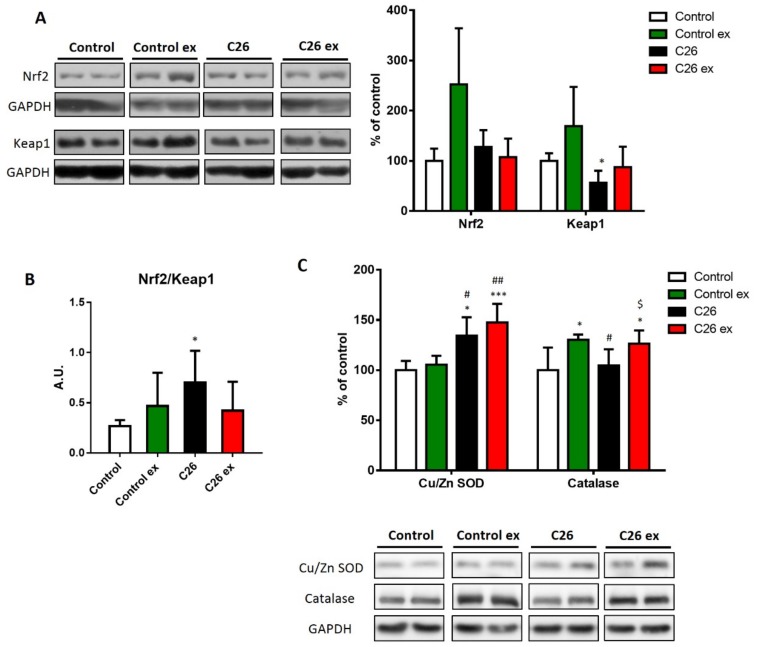

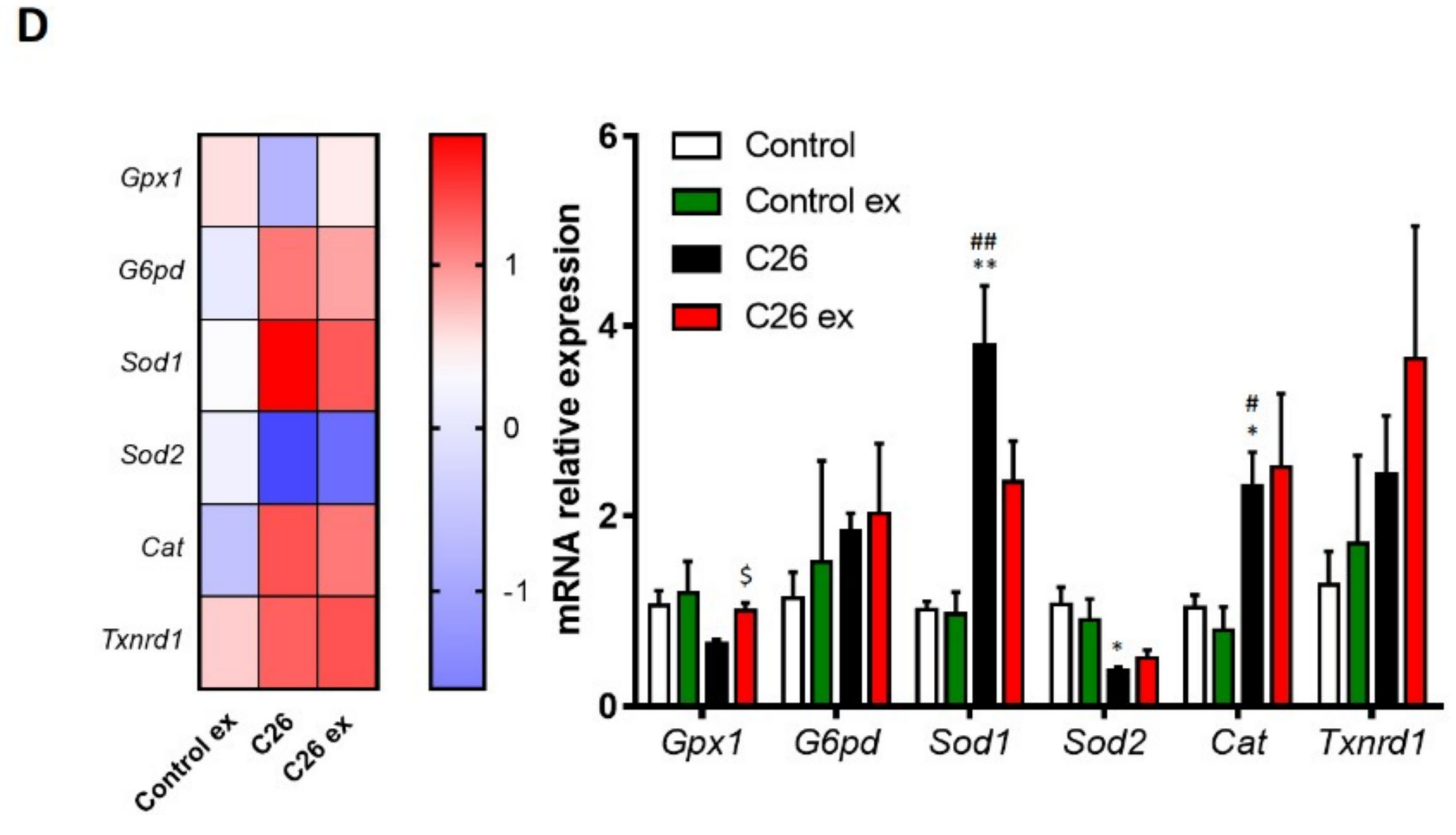
Antioxidant systems are upregulated in both sedentary and exercised tumor-bearing mice. Nrf2 and Keap1 protein levels (**A**; representative blot and quantification), Nrf2/Keap1 ratio (**B**), CuZn SOD and catalase protein levels (**C**; representative blot and quantification) and gene expression of antioxidant enzymes (**D**; heat map and histogram) of control (*n* = 5), control exercised (control ex; *n* = 6) and tumor-bearing mice either sedentary (C26; *n* = 8) or exercised (C26 ex; *n* = 8). Protein expression data (means ± SD) are percentage of control values. Nrf2/Keap1 ratio (means ± SD) is expressed as arbitrary units (A.U.). Gene expression data (means ± SEM) are ΔΔ ct and relative expression to control, respectively for heat map and histogram. Significance of the differences: * *p* < 0.05, ** *p* < 0.01, *** *p* < 0.001 vs. control; ^#^ *p* < 0.05, ^##^ *p* < 0.01 vs. control ex; ^$^ *p* < 0.05 vs. C26.

Oxidative balance is maintained by the intracellular antioxidant systems, that are mainly regulated by the transcriptional factor Nrf2 and by its inhibitor Keap1 [32]. In sedentary C26-bearing mice, Nrf2 was not affected, whereas Keap1 levels decreased (Figure 4A), resulting in increased Nrf2/Keap1 ratio (Figure 4B) that is suggestive of antioxidant response activation. In exercised tumor hosts, the levels of Nrf2 and Keap1 as well as their ratio did not change (Figure 4A,B). Although the differences were not significant, exercised healthy mice showed an increasing trend in both Nrf2 and Keap1 levels (Figure 4A), resulting in a Nrf2/Keap1 ratio comparable to control values (Figure 4B). To further investigate the antioxidant response, the protein levels of CuZn SOD, catalase and the gene expression of different antioxidant enzymes were analyzed (Figure 4C,D). In C26-bearing mice, the alteration of the redox balance was associated with increased CuZn SOD levels, with no changes of catalase protein expression (Figure 4C). The increase in CuZn SOD was coupled with upregulation of catalase levels in exercised C26-bearing mice as compared to both sedentary controls and tumor bearers (Figure 4C). In healthy animals, exercise was unable to modify the levels of CuZn SOD, while significantly increased catalase content (Figure 4C). At the transcript level, *Sod1* and *Cat* increased in C26-bearing mice, whereas *Sod2* was downregulated (Figure 4D). In exercised tumor hosts, *Gpx1* expression increased as compared to sedentary tumor bearers (Figure 4D), while the majority of the antioxidant genes analyzed showed an increasing, although not significant, trend that was not observed in healthy exercised animals (Figure 4D).

**Figure 5:**
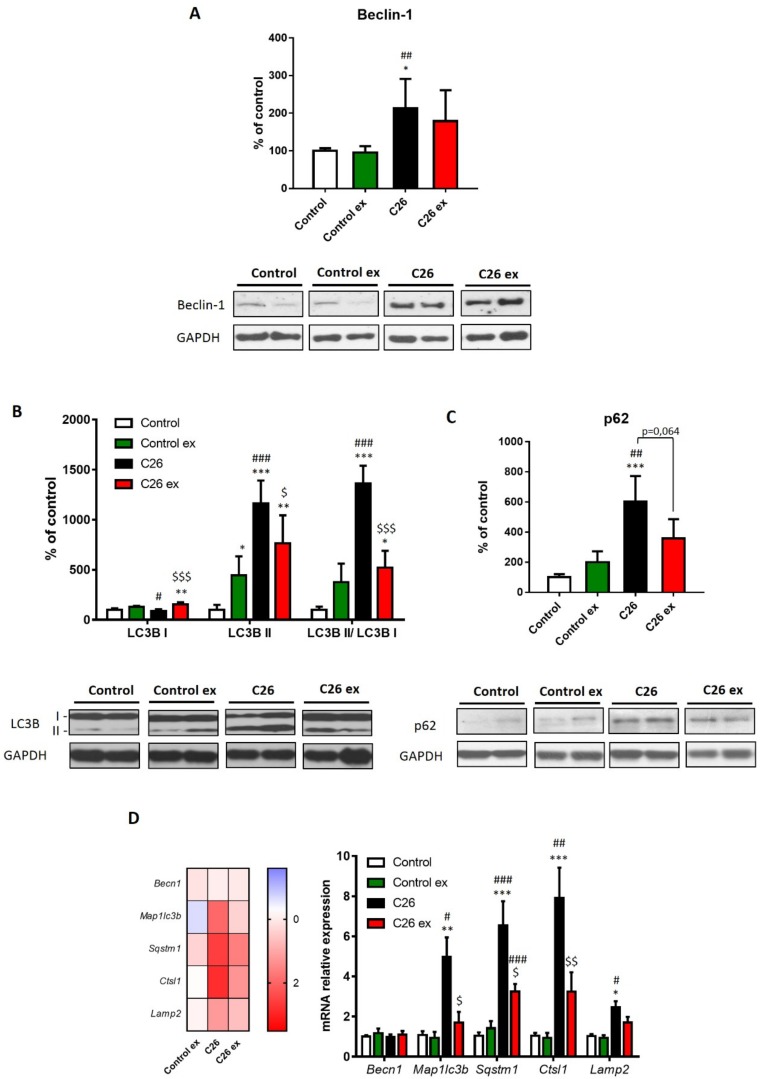
Exercise reduces the expression of markers of autophagy in tumor-bearing mice. Beclin-1 (**A**), LC3B (**B**) and p62 protein levels (**C**; representative blot and quantification) and gene expression of autophagy markers (**D**; heat map and histogram) of control (*n* = 5), control exercised (control ex; *n* = 6) and tumor-bearing mice either sedentary (C26; *n* = 8) or exercised (C26 ex; *n* = 8). Protein expression data (means ± SD) are expressed as percentage of control. Gene expression data (means ± SEM) are expressed as ΔΔ ct and relative expression to control, respectively for heat map and histogram. Significance of the differences: * *p* < 0.05, ** *p* < 0.01, *** *p* < 0.001 vs. control; ^#^ *p* < 0.05, ^##^ *p* < 0.01, ^###^ *p* < 0.001 vs. control ex; ^$^ *p* < 0.05, ^$$^ *p* < 0.01, ^$$$^ *p* < 0.001 vs. C26.

The reduction of carbonylated proteins occurring in exercised C26 hosts could derive from decreased substrate carbonylation but also from increased disposal of altered protein. In this regard, cancer-induced muscle wasting is well known to be associated with a protein hypercatabolic response [4]. Consistently with the loss of muscle mass, the gene expression of molecules accepted as markers of proteasome activation, namely *Fbxo32* and *Trim63*, was comparably upregulated in both sedentary and exercised tumor hosts, even though the difference in the latter did not reach the statistical significance (Figure S1). In line with previous observations [33], data reported in Figure 5 show that autophagy was induced in the muscle of C26-bearing mice. Notably, the levels of accepted markers such as beclin-1, the autophagosome-associated LC3B isoform II and the LC3B II/I ratio were upregulated (Figure 5A,B). Such a pattern was associated with increased levels of p62/SQSTM1, as a measure of substrate sequestration into autophagosomes (Figure 5C). Also, the mRNA levels of *Map1lc3b* and *Sqstm1* were upregulated in tumor hosts, together with increased *Ctsl1* and *Lamp2* gene expression (Figure 5D). Consistently with previous observations [33], no changes were observed as for *Becn1* expression (Figure 5D). In exercised C26-bearing mice, beclin-1 protein expression did not differ significantly as compared to control animals. Moreover, exercise reduced LC3BII content, the LC3BII/LC3BI ratio (Figure 5B) and p62/SQSTM1 levels in tumor-bearing animals (Figure 5C), although this latter did not reach the statistical significance, likely in view of the high intragroup variability. The same trend can also be observed for *Map1lc3b, Sqstm1* and *Ctsl1* transcript levels, that were reduced in exercised tumor hosts, while no effect was detectable for *Lamp2* mRNA levels (Figure 5D). As for healthy mice, exercise increased LC3BI levels above those of sedentary controls, without affecting the other markers of autophagy (Figure 5).

**Figure 6:**
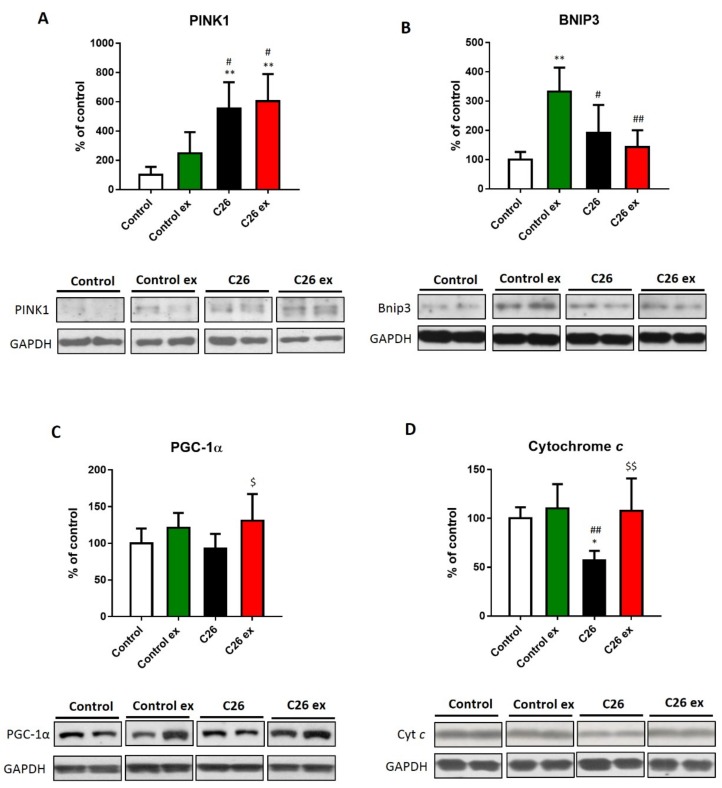
Exercise increases mitochondrial mass without affecting the expression of markers of mitophagy. PINK1 (**A**), BNIP3 (**B**), PGC-1α (**C**) and cytochrome c (**D**; representative blot and quantification) protein levels of control (*n* = 5), control exercised (control ex; *n* = 6) and tumor-bearing mice either se. dentary (C26; *n* = 8) or exercised (C26 ex; *n* = 8). Data (means ± SD) are expressed as percentage of control. Significance of the differences: * *p* < 0.05, ** *p* < 0.01 vs. control; ^#^ *p* < 0.05, ^##^ *p* < 0.01 vs. control ex; ^$^ *p* < 0.05, ^$$^ *p* < 0.01 vs. C26.

The maintenance of a redox homeostasis is also relevant to mitochondrial health. In this regard, oxidative stress may damage mitochondria and increase their degradation through mitophagy [19]. The present study demonstrated that in C26 tumor bearers the mitophagy marker PINK1 increased (Figure 6A), whereas the other mitophagy-related protein BNIP3 was not significantly affected, although it showed an increasing trend (Figure 6B). In the C26-bearing mice, exercise did not change PINK1 levels as compared to sedentary tumor hosts and BNIP3 levels remained comparable to controls (Figure 6A,B). The above results suggest that mitophagy could be increased in the C26 hosts. Along this line, in a physiologically regulated system, the enhancement of mitochondria disposal should induce mitochondrial biogenesis. To investigate this point, the levels of PGC-1α and cytochrome c, respectively markers of mitochondrial biogenesis and mass, were evaluated. In the C26-bearing mice, PGC-1α expression did not change (Figure 6C), whereas cytochrome c levels decreased as compared to controls (Figure 6D). On the other hand, both markers were higher in exercised than in sedentary tumor-bearing mice (Figure 6C,D). In healthy mice, exercise increased BNIP3 levels (Figure 6A), with no changes in both PGC-1α and cytochrome c levels (Figure 6C,D).

To better understand the relevance of oxidative stress in cachectic cancer patients, the ‘standard’ C26 model was modified adding the administration of chemotherapy, in order to generate an experimental condition more closely resembling the human pathology. Along this line, tumor-bearing mice were treated with oxaliplatin and 5-fluorouracil (C26 OXFU; see Methods for details and experimental schedule). The loss of muscle mass was higher in chemotherapy-treated than untreated tumor hosts (Figure S2A), likely due to both increased experimental period (28 days compared to 13 days of tumor growth, for C26 OXFU and C26, respectively) and drug administration. Exercise was able to improve muscle wasting in C26 OXFU mice (C26 OXFU = 89.2 ± 9.66, C26 OXFU ex = 117.2 ± 26.1 mg of tibialis anterior/initial body weight, *n* = 5 for both groups). Severe muscle wasting was coupled to increased protein carbonylation in C26 OXFU mice as compared to controls, OXFU and untreated tumor hosts (Figure S2B). The induction of oxidative stress in C26 OXFU was also coupled to increased G6PD activity (Figure S2C) and ROS levels (Figure S3A). Both the increased ROS (Figure S3A) and protein carbonylation (C26 OXFU = 9.1 ± 0.3, C26 OXFU ex = 9.6 ± 2.7 A.U., *n* = 5 for both groups) levels were not corrected in trained C26 OXFU animals. Of interest, the expression levels of Nrf2 and Keap1, lower than controls in sedentary C26 OXFU, was significantly increased by exercise (Figure S3B). Despite these modulations, the Nrf2/Keap1 ratio showed only an increasing trend in sedentary C26 OXFU mice, while the increase vs. control animals became significant in the C26 OXFU ex group (Figure S3C). However, no differences could be observed when C26 OXFU and C26 OXFU ex were compared (Figure S3C).

## 3. Discussion

Cachexia negatively impinges on the management of cancer patients, impairing the effectiveness of antineoplastic drugs and reducing patient survival and quality of life [3]. Despite being a relevant clinical issue, an effective treatment is still lacking. In this regard, different pharmacological approaches designed to counteract cancer-induced muscle wasting are under investigation, including anti-inflammatory drugs, inhibitors of muscle catabolic pathways and anabolism stimulating strategies [21]. Promising results came out also from exercise interventions, which are both cost-effective and multi-target [34]. Along this line, different exercise protocols enhanced muscle mass and function in experimental cancer cachexia [6,25], together with protection against mitochondrial alterations and increased protein degradation [6,8,35]. Notably, whereas resistance exercise results in a hypertrophic muscle phenotype, endurance/aerobic exercise is prone to muscle and systemic metabolic adaptations [36]. Consistently, moderate aerobic exercise performed on a treadmill well before tumor implantation was shown to improve muscle oxidative metabolism in the C26-bearing mice [6]. However, when animal training started after tumor implantation, body and muscle wasting was not counteracted [6], focusing the attention on the issue of exercise timing and intensity in tumor hosts. In the present study, a different modality of exercise was tested, letting the animals run on a motorized wheel. In comparison with the treadmill apparatus, this setting allowed us to reduce the speed and to increase the running slope, likely enhancing the resistance component of training. Endurance and resistance exercise may activate distinct molecular pathways due to the different frequency and mechanical load imposed to the muscle [37]. Indeed, endurance exercise results in different metabolic adaptations leading to increased mitochondrial mass, oxygen delivery, glucose uptake and antioxidant capacity, whereas resistance exercise mainly leads to increased muscle mass [37]. For these reasons, coupling endurance and resistance exercise could result in good outcomes in cancer hosts, as shown by different pre-clinical and clinical studies [38,39,40]. Consistently, the protocol used in the present study exerted beneficial effects on the C26-bearing mice, as demonstrated by the increase of muscle mass and strength in comparison with sedentary tumor hosts. By contrast, exercise did not impinge on body and muscle parameters in healthy mice, likely due to the moderate intensity of the protocol. 

Among the several proposed molecular mechanisms, also oxidative stress could participate to the development of muscle wasting [9]. In both experimental and human cancer cachexia, markers of oxidative stress were upregulated, together with altered antioxidant response [16]. The present study confirmed the occurrence of oxidative stress in the muscle of the C26 hosts, since ROS and GSH levels were altered, coupled with an increase of the GSSG/GSH ratio. However, the amount of oxidized proteins and lipids in the skeletal muscle were not increased, in agreement with previous data obtained in the same experimental model [29]. The lack of correlation among markers of oxidative stress and oxidized products could depend on the increased disposal of damaged proteins; this could reflect the enhanced protein breakdown rates occurring in the muscle of tumor-bearing animals [4]. In this regard, several studies showed that oxidative stress modulates autophagy and enhances proteasome-dependent degradation [14,16]. Notably, oxidative stress was associated with increased levels of different markers of autophagy in mice lacking SOD1 in the skeletal muscle [41] as well as in murine myotubes exposed to H_2_O_2_ [42]. Therefore, the increase in proteolysis induced by oxidative stress could be a mechanism for the disposal of oxidized cellular products. In line with previous observations [33,43], C26-bearing mice showed an upregulation of Trim63, *Fbox32* and proteins considered as markers of autophagy. Therefore, besides inducing muscle wasting, the upregulation of both proteasome-dependent and autophagic degradation likely contributed to normalizing the levels of oxidized products in cachectic muscles.

Most of pre-clinical and clinical studies on cancer cachexia reported an upregulation of antioxidant enzymes, suggesting that the redox imbalance was likely generated by ROS overproduction rather than by impaired antioxidant capacity. In particular, CuZn SOD and catalase expression were enhanced in the skeletal muscle of tumor-bearing animals [11,29] and, similarly, cancer patients showed increased antioxidant enzyme levels and activity [15]. Consistently, the present study showed that the antioxidant response was activated in the muscle of C26-bearing mice, even if such activation was not able to restore the tissue redox potential. The antioxidant response is mainly regulated by the transcription factor Nrf2 [32]. In line with the antioxidant enzyme expression, in the present study tumor hosts showed increased Nrf2/Keap1 ratio, likely induced by muscle oxidative stress. In this regard, enhanced Nrf2 signaling was reported in pathological conditions such as AVMs; this disease is characterized by disrupted autophagic flux, as suggested by the accumulation of autophagy-related markers, that eventually leads to dysregulation of the cellular redox homeostasis [44]. Of interest, in addition to Keap1-dependent engagement, Nrf2 can also be activated through another pathway mediated by p62/SQSTM1 [44]. Therefore, the enhanced Nrf2 signaling shown in sedentary tumor hosts could also derive from p62 accumulation.

The redox balance is positively modulated by continuous and non-strenuous exercise [27]. In this regard, exercise was reported to reduce the levels of markers of oxidative stress in healthy rats exposed to chemotherapy [45], in heart failure-induced myopathy [46] and in rats bearing the Walker-256 tumor [47]. In the present study, exercise decreased protein carbonylation in the C26-bearing mice, while MDA levels were not affected. Such reduction could derive from both increased muscle antioxidant capacity and efficient disposal of autophagosomes, this latter resulting in reduced amount of damaged proteins. These findings are consistent with previous reports demonstrating that voluntary running in tumor hosts was able to improve muscle wasting by down-regulating autophagy [25]. Along this line, the reduction of ROS levels and the unchanged Nrf2/Keap1 ratio observed in the skeletal muscle of exercised C26-bearing mice could reflect a partial restoration of normal autophagy and redox homeostasis. However, the observation that GSH levels remained altered with respect to control values could suggest that exercise did not completely revert the redox imbalance in tumor hosts. Although several studies previously reported the antioxidant effects of exercise [27], the training protocol presented in this study did not considerably impinge on the muscle redox balance in healthy mice, with the exception of an upregulation of catalase levels. Such discrepancy could rely on the type (moderate) and timing of exercise adopted in the present study, as suggested by Oh-ishi et al. [48]. However, an increasing trend can be observed in both Nrf2 and Keap1 protein expression without changes in the Nrf2/Keap1 ratio. This observation could suggest that in the experimental conditions adopted in the present study, exercise in healthy animals could act as a sort of preconditioning, increasing the amount of Nrf2 that could be readily available to face the occurrence of an oxidative damage.

Cancer cachexia is also associated with perturbations of the mitochondrial compartment, in particular altered mitochondrial biogenesis, function and disposal [6,8]. Notably, mitochondrial perturbations trigger ROS emission and could result in mitophagy upregulation [5]. Emerging evidence show that mitochondrial degeneration plays an important role in the development of muscle wasting in cachectic animals [5]. Consistently, the present study revealed that sedentary C26-bearing mice showed increased levels of PINK1, a marker of mitophagy, that were not modified by exercise. In addition, damaged mitochondria were not efficiently replaced in sedentary tumor hosts, as suggested by the reduced muscle cytochrome c content. Among the different targets, aerobic exercise also impinges on mitochondrial homeostasis, increasing mitochondrial number, function and muscle oxidative capacity [49]. Although the increase in PINK1 expression is not corrected, exercise in C26-bearing mice increases the expression of markers of mitochondrial biogenesis and mass, suggesting an effective turnover of these organelles. Consistently with previous observations [50], exercise reveals to be able to marginally affect both autophagy and mitophagy, as demonstrated by the increased levels of LC3BII and BNIP3. 

Oxidative stress could also be enhanced by chemotherapeutic treatment in cachectic patients [51]. Alterations in the redox balance were found in healthy chemotherapy-treated animals, in which oxidative stress resulted in increased oxidized products, proteolysis and muscle wasting [45,52]. The present study shows that the combination of tumor and chemotherapy (C26 OXFU) leads to increased protein carbonylation that was associated with enhanced G6PD activity, likely due to an adaptive, though unsuccessful, response of the muscle to face the redox imbalance. Although exercise exerts positive effects on the redox balance of tumor-bearing mice, the same protocol improves muscle wasting but not oxidative stress in chemotherapy-treated C26 hosts. The severity of the model as well as the end-point chosen for animal sacrifice could have masked the beneficial effects of exercise in terms of redox homeostasis. On the other side, the improvement of muscle wasting exerted by exercise suggests that the super-imposition of chemotherapy in a tumor-bearing organism might lead to the activation of mechanisms of tissue depletion that are only marginally influenced by oxidative stress. 

## 4. Materials and Methods

### 4.1. Reagents

All the materials were supplied by MilliporeSigma (Burlington, MA, USA), unless differently specified.

### 4.2. Animals and Experimental Design

Experimental animals were cared for in compliance with the Italian Ministry of Health Guidelines (n°63/2014-B; 20/02/2014) and the Policy on Humane Care and Use of Laboratory Animals (NRC 2011). The experimental protocol was approved by the Bioethical Committee of the University of Torino. Male BALB/c mice (6-week-old; Charles River, Wilmington, MA, USA) were maintained on a 12:12 hour dark-light cycle with controlled temperature (from 18 to 23 °C) and free access to food (Global Diet 2018, Mucedola, Settimo Milanese, Italy) and water during the whole experimental period.

Mice received a single subcutaneous injection between the shoulder blades with 5 × 10^5^ C26 colon carcinoma cells obtained from Prof. Mario P. Colombo (Milano, Italy) and originally characterized by Corbett et al. [53]. C26 cells were maintained in vitro in DMEM supplemented with 10% FBS, 100 U/mL penicillin, 100 µg/mL streptomycin, 100 µg/mL sodium pyruvate, 2 mM L-glutamine, at 37 °C in a humidified atmosphere of 5% CO_2_ in air. The day of tumor implantation, C26 cells were detached with trypsin, resuspended in sterile saline and implanted in the back of the animals. Mouse weight and food intake were recorded daily, starting from the day of tumor implantation. Animals were sacrificed under anesthesia with Avertin (2,2,2-tribromoethanol, Tert-amyl alcohol; kept in dark and at 4 °C; 300 mg/kg) in different days specified in the experimental protocols (see below). Muscles and tissues were rapidly excised, weighed, frozen in liquid nitrogen and stored at −80 °C for further analysis.

Mice were randomized according to body weight and divided into two groups, namely controls (*n* = 11) and C26 colon carcinoma bearers (C26, *n* = 16). Both controls and C26 were further divided into two sub-groups (*n* = 5/6 for controls and *n* = 8 for C26), sedentary or exercised. As for the exercise protocol, we adapted the intensity and the schedule of the treadmill exercise presented in Pin et al. [6] to a custom motorized wheel (radius = 16 cm). Using the motorized wheel with a moderate speed allowed a mild intensity aerobic exercise and increased the resistance component of training due to the hill as compared to the flat treadmill, resulting in a mixed endurance-resistance exercise. Mice were adapted to the motorized wheel for 5 days before tumor injection, starting from 5 m/min for 15 min and increasing speed and time daily until reaching 11 m/min for 45 min. Starting on the day after tumor implantation, animals were exercised 3 days out of 4 at 11m/min for 45 min until the day before sacrifice (12 days after tumor transplantation). Mice were exercised during the light cycle at Zeitgeber time 7 (ZT7, ZT0 is lights-on and ZT12 is lights-off time). The daily exercise session was interrupted for single animals when they gave evidences of exhaustion. Overall, the majority of tumor-bearing mice completed the exercise protocol. All the animals were sacrificed 13 days after tumor transplantation. As for exercised control and C26-bearing mice, sacrifice occurred at least 24 h after the last exercise session.

For the chemotherapy experiment, the following regimen was adopted: oxaliplatin (OX, 6 mg/kg, i.p.; Accord Healthcare, UK) followed (2 h later) by 5-fluorouracil (FU, 50 mg/kg, i.p.; Accord Healthcare, UK). The chemotherapeutic regimen was based on previously published reports [54,55,56] and on our preliminary studies, in order to prolong tumor-bearing mice survival. The dose was adjusted for actual body weight. Before starting the experiment, animals were randomized according to body weight and divided into four groups, namely controls (*n* = 5), healthy chemotherapy-treated mice (OXFU, *n* = 6), C26 colon carcinoma bearers (C26, *n* = 8) and C26 colon carcinoma bearers treated with chemotherapy (C26 OXFU, *n* = 8). Mice received OXFU weekly, starting 7 days after tumor injection. C26 mice were sacrificed at day 14 whereas controls, OXFU mice and C26 OXFU mice were sacrificed 28 days after tumor implantation. The choice of the different sacrifice time-points was based on a preliminary experiment aimed to identify the occurrence of the humane termination end-points. In particular, the end-point criteria combined the body mass loss and the evaluation of mouse appearance and posture (lack of grooming, piloerection, and hunched posture), natural and provoked behavior (inactivity, impaired locomotion, and reduced reactivity to external stimuli), and food intake/ability to eat and drink. 

For the exercise experiment on C26 OXFU mice, animals were randomized according to body weight and divided into three groups, namely controls (*n* = 5), sedentary C26 OXFU (*n* = 5) and exercised C26 OXFU (*n* = 5).The adaptation and exercise protocol for C26 OXFU mice was the same as for C26 animals (see above) but prolonged until the day before sacrifice (27 days after tumor transplantation; see also Ballarò et al. [30]. The weekly schedule was 2 days exercise, 1 day rest (chemotherapy administration), 3 days exercise and 1 day rest. Exercise time and adherence were the same as reported above for untreated C26-bearing mice. Controls, sedentary and exercised C26 OXFU mice were euthanized 28 days after tumor transplantation.

### 4.3. Grasping Test

Muscle strength was assessed by the grasping test as previously described in [6] using a Panlab-Harvard Apparatus device, with slight modifications. Mice were positioned on the grid connected to a dynamometer posing both forelimbs and hindlimbs and measurements were recorded after pulling the animal’s tail. The measurements were done in triplicate for each mouse before tumor implantation and at day 13 of tumor growth. 

### 4.4. Western Blotting

Approximately 20 mg of gastrocnemius muscle were homogenized in 10 mM Tris (pH 7.5), containing 0.25 M sucrose, 50 mM NaCl, 5 mM EDTA, 30 mM sodium pyrophosphate, 1% Nonidet P-40, 0.25% sodium deoxycholate, 50 mM NaF, 0.1 mM sodium orthovanadate with freshly added protease inhibitor cocktail, sonicated and incubated on ice for 30 min, centrifuged at 12,000× *g* for 12 min at 4 °C, and the supernatant collected as whole-cell extract. Protein concentration was measured according to Lowry’s, using BSA as working standard. Equal amounts of total extract protein (from 20 to 30 µg) were heat-denaturated in sample-loading buffer (50 mM Tris-HCl, pH 6.8, 100 mM dithiothreitol, 2% SDS, 0.1% bromophenol blue, 10% glycerol), resolved by SDS-PAGE and transferred to polyvinylidene difluoride membranes. The filters were blocked with Tris-buffered saline (TBS) containing 0.05% Tween and 5% BSA and then incubated overnight with antibodies (diluted in TBS containing 0.05% Tween and NFDM or BSA depending on manufacturer’s instructions) directed against Nrf2 (sc-722, Santa Cruz Biotechnology, Dallas, TX, USA), Keap1 (sc-33569, Santa Cruz Biotechnology, Dallas, TX, USA), CuZn SOD (SOD-100; Assay Designs, Ann Arbor, MI, USA), catalase (C 0979), beclin-1 (B6186), LC3B (L7583), p62 (5114; Cell Signaling Technology Inc., Beverly, MA, USA), PGC-1α (AB3242; Millipore, Temecula, CA, USA), cytochrome c (556433; BD Biosciences, Franklin Lakes, NJ, USA), BNIP3 (ab38621; Abcam, Cambridge, UK), PINK1 (SAB2500794). Peroxidase-conjugated IgG (Bio-Rad, Hercules, CA, USA) were used as secondary antibodies. Membrane-bound immune complexes were detected by enhanced chemiluminescence (Amersham, Bio-Rad, Hercules, CA, USA) with a camera imaging system (Imagequant LAS 4000, GE Healthcare, Little Chalfont, UK). Protein loading was normalized according to GAPDH (G9545) expression. Band quantification was performed by densitometric analysis using the TotalLab software (NonLinear Dynamics, Quayside, UK).

### 4.5. ROS Levels

ROS were measured as described in reference [57], with slight modifications. Briefly, gastrocnemius total protein extracts were incubated with 10 µl of 2′,7′-dichlorofluorescin diacetate (DCFH-DA; 5 µM) for 20 min at 37 °C. DCFH-DA is a stable, non-fluorescent molecule that readily crosses the cell membrane and is hydrolyzed by intracellular esterase to non-fluorescent 2′,7′-dichlorofluorescin (DCFH). DCFH is rapidly oxidized in the presence of peroxides to the highly fluorescent 2′,7′-dichlorofluorescein (DCF). Fluorescence (excitation/emission at 485/538 nm) was measured on a 96-well microplate reader. The results were normalized to total protein content from an aliquot of the muscle homogenate, assessed according to Bradford, with bovine serum albumin as standard.

### 4.6. Glutathione Levels

Glutathione was determined as described in reference [57], with slight modifications. Briefly, an aliquot of gastrocnemius total protein extract was used to quantify GSH and GSSG. Samples were deproteinized using 5% metaphosphoric acid on ice, centrifuged at 15,000× *g* for 2 min. Supernatants were treated with 4 M triethanolamine to reach pH 7.4. GSH concentration was determined after 2 minutes incubation with 5,5′-dithiobis-2-nitrobenzoic acid (DTNB) by measuring the production of 5′-thio-2-nitrobenzoic acid (TNB) at 412 nm on a 96-well microplate reader. Suitable volumes of diluted glutathione reductase (6 U/mL) and of NADPH (4 mg/mL) were then added to evaluate total glutathione level (GSH + GSSG). GSSG content was calculated by subtracting GSH content to total glutathione levels. The results were normalized to total protein content from an aliquot of the muscle homogenate, assessed according to Bradford, with bovine serum albumin as standard.

### 4.7. G6PD Enzymatic Activity

G6PD activity was determined as described in reference [58]. Briefly, approximately 50 mg of gastrocnemius muscle were homogenized in 0.1 M triethanolamine, 7 mM ClMg_2_, (pH 7.6) with freshly added protease inhibitor cocktail. Samples were sonicated and centrifuged at 10,000× *g* for 15 min at 4 °C. In order to evaluate the G6PD activity, 10 mM glucose-6-phosphate in potassium phosphate buffer and 400 μL of gastrocnemius homogenate were added to a cuvette. The reaction was initiated by adding 0.9 mM of NADP to the cuvette. The absorbance was read over 3 min at 340 nm using a spectrophotometer. The results were normalized to total protein content from an aliquot of the muscle homogenate, determined according to Bradford, with bovine serum albumin as standard.

### 4.8. Protein Carbonylation Assay

Oxidative modification of total proteins was assessed by immunoblot detection of muscle protein carbonyl groups using the OxyBlot Protein Oxidation kit in accordance with the manufacturer’s instructions (Millipore, Temecula, CA, USA). About 20 mg of total protein extract from gastrocnemius were derivatized by reacting with 2,4-dinitrophenylhydrazine so that they could be detected by western blotting using specific antibodies. After derivatization, samples were resolved by SDS-PAGE, transferred to polyvinylidene difluoride membranes and incubated with the anti-dinitrophenylhydrazone (DNP) antibody (57150; Intergen Company, Purchase, NY, USA). Peroxidase-conjugated IgG (Bio-Rad, Hercules, CA, USA) were used as secondary antibodies. Membrane-bound immune complexes were detected by enhanced chemiluminescence (Amersham, Bio-Rad, Hercules, CA, USA) with a camera imaging system (Imagequant LAS 4000, GE Healthcare, Little Chalfont, UK). Protein loading was normalized according to Ponceau Red staining. Band quantification was performed by densitometric analysis using the TotalLab software (NonLinear Dynamics, Quayside, UK).

### 4.9. MDA Quantitation

MDA levels were determined as described in [58]. Briefly, approximately 20 mg of gastrocnemius muscle were homogenized in 50 mM potassium phosphate buffer, containing 1 mM EDTA, pH 7.4. Samples were sonicated, incubated on ice for 30 min and then centrifuged at 500× *g* for 5 min at 4 °C. The determination of MDA levels in muscle homogenates is based on the hydrolysis of lipid peroxides and subsequent formation of the adduct thiobarbituric acid (TBA) and MDA (TBA–MDA_2_). This adduct was detected by reverse phase High Performance Liquid Chromatography (HPLC) and quantified at 532 nm (Ultimate 3000 Dionek; Thermo Fisher, Waltham, MA, USA). The chromatographic technique was performed in isocratic mobile phase being a mixture of 50 mM KH_2_PO_4_ (pH 6.8) and acetonitrile (70:30). The results were normalized to total protein content from an aliquot of the muscle homogenate, determined by Bradford’s method with bovine serum albumin as standard.

### 4.10. Real-Time PCR

Total RNA was obtained from tibialis muscles using the TRI Reagent following manufacturer’s instructions. RNA concentration was determined fluorometrically using the Ribogreen reagent (Invitrogen, Carlsbad, CA, USA). Total mRNA was retro-transcribed using the i-Script cDNA synthesis kit (Bio-Rad, Hercules, CA, USA). Transcript levels were determined by real-time PCR using the SsoAdvanced SYBR Green Supermix and the CFX Connect Real-Time PCR Detection System (Bio-Rad, Hercules, CA, USA). Primer sequences are given in the Supplemental Material section (Table S1).

### 4.11. Statistical Analysis

All the results are expressed as means ± SD, except where otherwise stated. Significance of the differences was performed using SPSS software (IBM, North Castle, NY, USA) evaluated by two-way analysis of variance (ANOVA) and by Tukey’s *post-hoc* test for multiple comparisons. Differences were considered significant when *p* < 0.05. 

## 5. Conclusions

The presence of the tumor resulted in redox imbalance in the skeletal muscle, likely contributing to the wasting pattern. However, the possible accumulation of oxidized products that derives from oxidative stress could be counterbalanced by the active catabolism in cachectic animals. In contrast, tumor and chemotherapy severely affected muscle metabolism, resulting in increased oxidative stress and protein carbonylation. Regarding untreated tumor-bearing mice, exercise attenuated muscle wasting and completely reversed the loss of muscle strength. The possible mechanism by which exercise relieved muscle wasting in tumor hosts could be related to improved redox balance, reduced accumulation of damaged proteins and an efficient mitochondrial turnover. Similarly to untreated tumor-bearing mice, exercise relieved muscle atrophy but did not impinge on muscle oxidative stress in chemotherapy-treated C26 hosts. Overall, these findings underline the relevance of moderate exercise to alleviating muscle oxidative stress and protein hypercatabolism in cancer hosts, also contributing to improve energy metabolism. However, the mechanisms by which exercise improves muscle wasting in C26-bearing mice exposed to chemotherapy might not be related to the restoration of muscle oxidative homeostasis.

## Supplementary Materials

The following are available online at https://www.mdpi.com/2072-6694/11/3/285/s1, Figure S1: Atrogene expression in sedentary and exercised tumor-bearing mice, Figure S2: Chemotherapy exacerbates muscle wasting and oxidative stress in tumor-bearing mice, Figure S3: Exercise does not considerably impinge on muscle redox balance in chemotherapy-treated tumor-bearing mice, Table S1: Forward and reverse primer sequences (5′-3′) of analyzed transcripts.

## Abbreviations

ROS	reactive oxygen species
PI3K	phosphoinositide 3-kinases
Akt	protein kinase B
mTORC1	mammalian target of rapamycin complex 1
MAPK	mitogen-activated protein kinase
AMPK	5′ AMP-activated protein kinase
FoxO	forkhead box O3
SOD	superoxide dismutase
GPxs	glutathione peroxidases
GSSG/GSH	oxidized/ reduced glutathione
NADP/NADPH	oxidized/ reduced nicotinamide adenine dinucleotide phosphate
G6PD	glucose-6-phosphate dehydrogenase
MDA	malonyldialdehyde
Nrf2	nuclear factor (erythroid-derived 2)-like 2
Keap1	kelch-like ECH-associated protein 1
Fbxo32	F-Box Protein 32
Trim63	tripartite motif containing 63
Sqstm1	sequestosome-1
Map1lc3b	microtubule-associated proteins 1A/1B light chain 3B
Becn1	beclin-1
Ctsl1	cathepsin L
Lamp2	lysosome-associated membrane protein 2
PINK1	PTEN-induced kinase 1
BNIP3	Bcl-2/adenovirus E1B 19-kDa interacting protein
PGC-1α	peroxisome proliferator-activated receptor gamma coactivator 1-alpha
OXFU	oxaliplatin and 5-fluorouracil
AVMs	autophagic vacuolar myopathies
DMEM	Dulbecco’s modified eagle medium
FBS	fetal bovine serum
BSA	bovine serum albumin
