# Supplementary material for: Moderate Exercise Improves Experimental Cancer Cachexia by Modulating the Redox Homeostasis
Source: Cancers (Basel). 2019 Feb 28;11(3):285. doi: 10.3390/cancers11030285 (PMC6468783; doi:10.3390/cancers11030285)
Supplement: Supplementary file 1 [file cancers-11-00285-s001.pdf]

Supplementary Materials: Moderate Exercise Improves Experimental Cancer Cachexia by Modulating the Redox Homeostasis

Riccardo Ballarò, Fabio Penna, Fabrizio Pin, Mari Carmen Gómez-Cabrera, José Viña and Paola Costelli

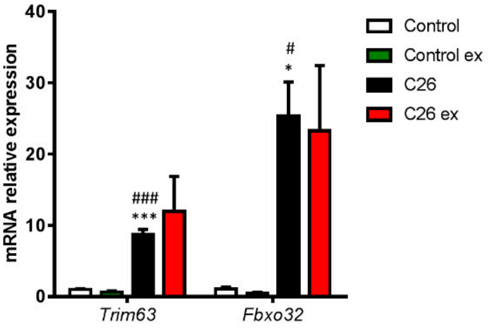

Figure S1. Atrogene expression in sedentary and exercised tumor-bearing mice. *Trim63* and *Fbxo32* gene expression of control (n = 5), control exercised (control ex; n = 6) and tumor-bearing mice either sedentary (C26; n = 8) or exercised (C26 ex; n = 8). Data (means ± SEM) are expressed as relative expression to control. mRNA levels are normalized to both *Actb* and *Tbp* gene expression. * $p < 0.05$, *** $p < 0.001$ vs. control; # $p < 0.05$, ### $p < 0.001$ vs. control ex.

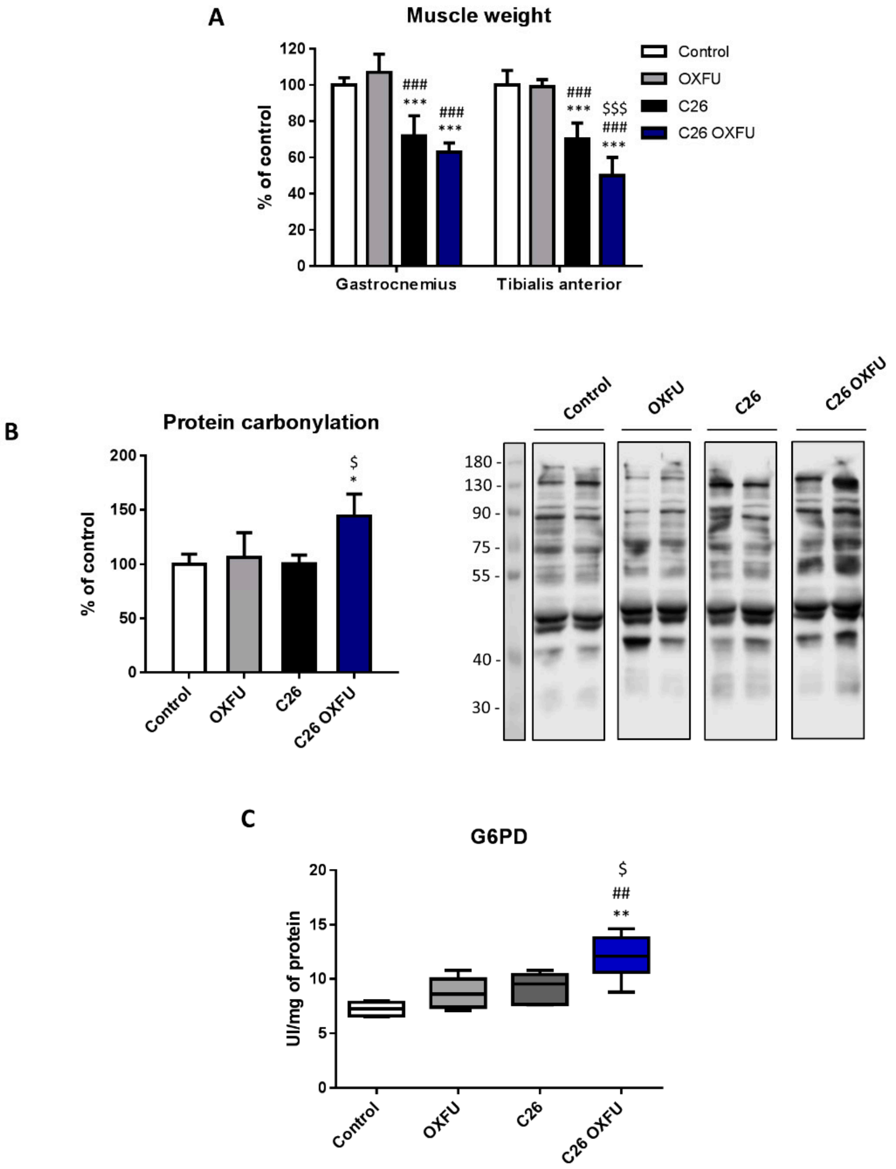

Figure S2. Chemotherapy exacerbates muscle wasting and oxidative stress in tumor-bearing mice. Muscle weight (**A**), protein carbonylation (**B**) representative blot and quantification) and G6PD enzymatic activity (**C**) of control ($n = 4$), healthy chemotherapy-treated mice (OXFU; $n = 5$) and tumor-bearing mice either untreated (C26; $n = 8$) or treated with chemotherapy (C26 OXFU; $n = 8$). Muscle weight and protein carbonylation levels (means ± SD) are expressed as percentage of control. G6PD activity (median and min to max whiskers) is expressed as the ratio between International Unit (IU) and mg of protein. Significance of the differences: * $p < 0.05$, ** $p < 0.01$, *** $p < 0.001$ vs. control; $^{\#\#}$ $p < 0.01$, $^{\#\#\#}$ $p < 0,001$ vs. control ex; $^{\$}$ $p < 0.05$, $^{\$\$\$}$ $p < 0.001$ vs. C26.

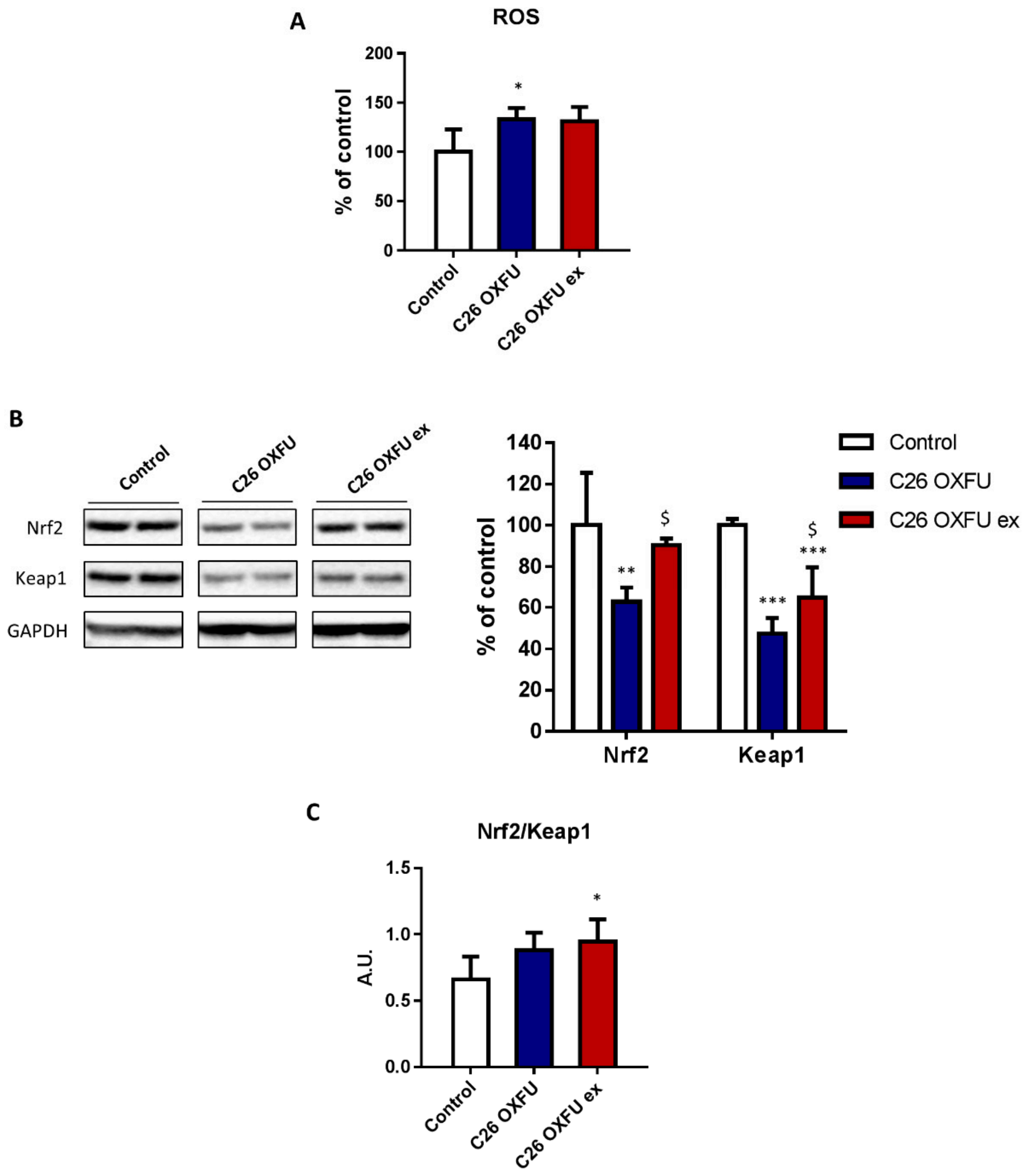

Figure S3. Exercise does not considerably impinge on muscle redox balance in chemotherapy-treated tumor-bearing mice. ROS levels (**A**), Nrf2 and Keap1 protein levels (**B**; representative blot and quantification), Nrf2/Keap1 ratio (**C**) of control (*n* = 5) and chemotherapy-treated tumor-bearing mice either sedentary (C26 OXFU; *n* = 5) or exercised (C26 OXFU ex; *n* = 5). Protein expression data and ROS (means ± SD) are percentage of control values. Nrf2/Keap1 ratio (means ± SD) is expressed as arbitrary units (A.U.). Significance of the differences: * $p < 0.05$, ** $p < 0.01$, *** $p < 0.001$ vs. control; $ $p < 0.05$ vs. C26 OXFU.

Table 1. Forward and reverse primer sequences (5′–3′) of analyzed transcripts.

Gene	Forward (5′–3′)	Reverse (5′–3′)
Tbp	TGCACAGGAGCCAAGAGTGAA	CACATCACAGCTCCCCACCA
Actb	CTGGCTCCTAGCACCATGAAGAT	GGTGGACAGTGAGGCCAGGAT
Gpx1	GTTTCCCGTGCAATCAGTTC	CATTCCGCAGGAAGGTAAAG
G6pd	CCGGAAACTGGCTGTGCGCT	CCAGGTCACCCGATGCACCC
Sod1	CCAGTGCAGGACCTCATTTT	TTGTTTCTCATGGACCACCA
Sod2	GGCCAAGGGAGATGTTACAA	GCTTGATAGCCTCCAGCAAC
Cat	AGCGACCAGATGAAGCAGTG	TCCGCTCTCTGTCAAAGTGTG
Txnrd1	CAGCGAGGAGACCATAGAGG	GCACATTGGTCTGCTCTTCA
Becn1	TGAAATCAATGCTGCCTGGG	CCAGAACAGTATAACGGCAACTCC
Map1lc3b	CACTGCTCTGTCTTGTGTA	TCGTTGTGCCTTTATTAGTG
Sqstm1	ATCGGAGGATCCGAGTGT	TGGCTGTGAGCTGCTCTT
Ctsl1	GTGGACTGTTCTCACGCTCAAG	TCCGTCCTTCGCTTCATAGG
Lamp2	GCTGAACAACAGCCAAATTA	CTGAGCCATTAGCCAAATACAT
Fbxo32	TCACAGCTCACATCCCTGAG	AGACTTGCCGACTCTTTGGA
Trim63	TGTCTGGAGGTCGTTTCCG	ATGCCGGTCCATGATCACTT

© 2019 by the authors. Licensee MDPI, Basel, Switzerland. This article is an open access article distributed under the terms and conditions of the Creative Commons Attribution (CC BY) license (http://creativecommons.org/licenses/by/4.0/).
